# Commentary: Eighteen cases of renal aneurysms: clinical retrospective analysis and experience of endovascular interventional treatment

**DOI:** 10.3389/fsurg.2024.1352880

**Published:** 2024-01-29

**Authors:** Som P. Singh, Ursula Qureshi, Farah Qureshi, Fawad Qureshi

**Affiliations:** ^1^ Department of Biomedical Sciences, Kansas City School of Medicine, University of Missouri, Kansas City, MO, United States; ^2^ College of Osteopathic Medicine, Kansas City University of Medicine and Biosciences, Kansas City, MO, United States; ^3^ Lake Erie College of Osteopathic Medicine, Erie, PA, United States; ^4^ Department of Nephrology and Hypertension, Mayo Clinic, Rochester, MN, United States

**Keywords:** renal artery aneurysm, endovascular, coil embolism, stenting, study design

A Commentary on Eighteen cases of renal aneurysms: clinical retrospective analysis and experience of endovascular interventional treatment By Lu T, Lin B, Zhang Y-p, Zhang J-h, Luo J-W, Tang Y, Fang Z-T. (2023). Front Surg. 10:1106682. doi:10.3389/fsurg.2023.1106682

## Introduction

Renal artery aneurysms account for approximately 1 in every 5 visceral aneurysms (1, 2). While these vascular malformations are often found incidentally on imaging, they can also symptomatically present with flank pain, hypertension, and their rupture can lead to fatal massive hemorrhage requiring immediate intervention (1, 3). Historically, open surgery was indicated for the management of renal artery aneurysms until the advent of interventional methodologies in the renal vasculature (3–5).

The growing use of these endovascular interventions have transformed both diagnostic and therapeutic patient care worldwide (1, 3). Among those with diseases of vascular etiology, endovascular interventions consistently demonstrate a lower mean cost per hospital admission compared to open surgical interventions (6). This understanding has been backed by years of clinical data that demonstrates the utility of endovascular interventions across numerous vascular pathologies (6, 7). However, there remains a paucity of this data comparably for endovascular interventions on renal artery aneurysms. The authors of this article aim to comment on a recent study by Lu et al. which provides a cohort of individuals undergoing management of renal artery aneurysms via the endovascular methodology (1).

## Summary

The retrospective study by Lu et al. demonstrated a cohort of 18 patients with a total of 23 renal artery aneurysms (1). Renal artery aneurysms were found to be asymptomatic. Among these 18 patients, 13 underwent endovascular intervention for 14 total renal artery aneurysms. There were 5 interventions of interest: stent implantation, coil embolization, parent artery embolization, stent-assisted coiling embolization, and liquid embolic agent embolization. Among interventions, the most used procedure was parent artery embolization (*n* = 4), followed by simple coil embolization (*n* = 3), and all other endovascular interventions respectively (*n* = 2). The average diameter of aneurysms was approximately 2.2 ± 1.5 cm. Additionally, among this cohort undergoing endovascular intervention, there were four patients which experienced what the study classified to be as mild complications within the context of pain. Follow-up was performed among these patients, and there was no noted technical stent displacement or aneurysm recurrence.

## Discussion

**Figure 1:**
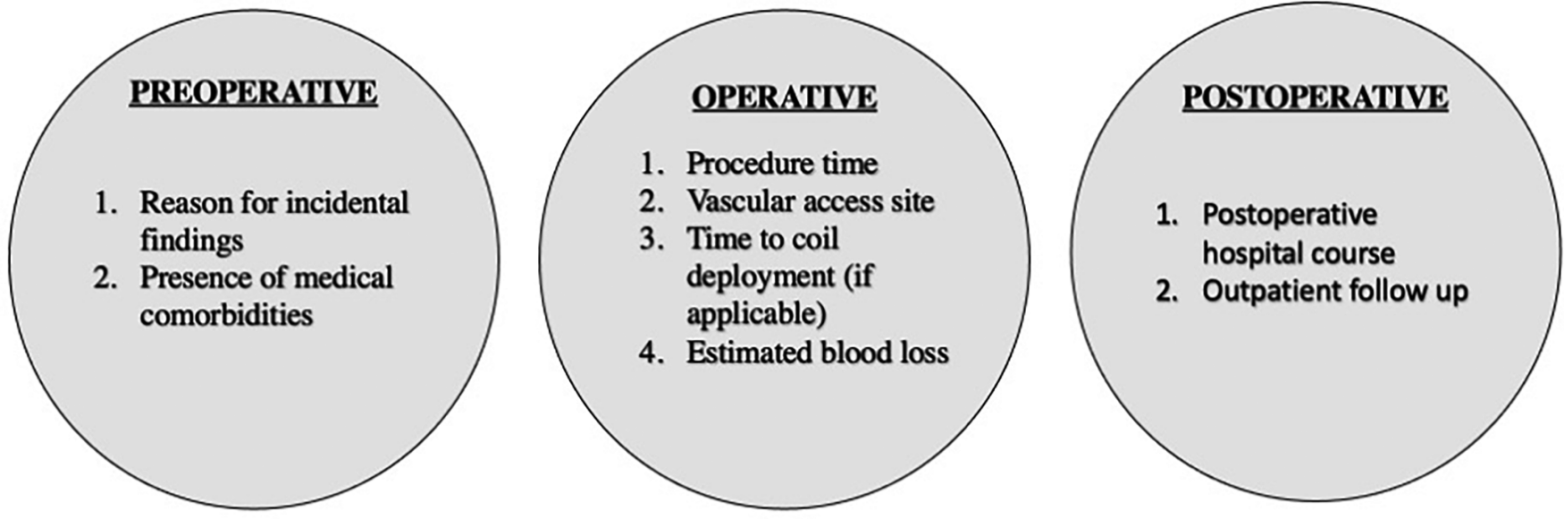
Proposed study variables for future clinical investigations at the preoperative, operative, and postoperative stages.

This study provides an overview of the interventional operations for renal artery aneurysm management. It formidably describes the utilization and recommended indication of five types of interventions. Beyond these descriptions, the authors of this commentary believe the data in this study can inspire a potential starting point for the discussion of future clinical investigations which compares the use of these various endovascular techniques. To the best of our knowledge, there are minimal clinical guideline recommendations that demonstrate an algorithmic approach to determine which intervention is best suited for a patient. Let alone, there remains a paucity of literature that effectively demonstrates the postoperative outcomes within each procedure. While this study does not directly compare the utility of the interventions (i.e., parent artery embolization vs. simple coil embolization), a secondary analysis of this cohort could be performed that specifically analyzed additional operative factors such as procedure time, access site, time to coil deployment, estimated blood loss, and post-operative hospital course (1, 8–10). This study proposal is shown in Figure 1.

In addition to the operative factors, this study characterizes some preoperative variables such as classifying whether the renal artery aneurysms were incidental finding on imaging or symptomatic. With regards to incidental findings, the authors of this commentary encourage future studies to further classify what the initial intervention was that led to the incidental finding. This can play a key role in determining the rationale behind the course of undergoing the endovascular operation (i.e., patient deemed high risk for open surgery) (8). Likewise, another study design could be to stratify pre-operative risk to create a risk calculator for endovascular renal artery aneurysm management (8–11).

Additionally, patients were followed in this study after procedural intervention, with some found to have been followed several years post-operation. This data in unclear regarding the number of follow up visits performed by each patient but does provide post-operative imaging results. In future studies, the post-operative setting could also provide the number of follow visits and be able to implement longitudinal variables that could be measured over a post-operative course (11–15). Although following a study of this label may require extensive patient adherence to follow up in its sample size.

## Conclusion

Overall, the study design by Lu et al. is sound in methodology and can be used as a framework for future clinical investigation. This commentary discusses suggests and discusses preoperative, operative, and postoperative variables that can be added to similar study designs. The goal of these variables is to generate a stronger clinical body of literature that can help clinicians and patients better understand and innovate endovascular intervention for renal artery aneurysms.
